# Functional Diversification of Thylakoidal Processing Peptidases in *Arabidopsis thaliana*

**DOI:** 10.1371/journal.pone.0027258

**Published:** 2011-11-07

**Authors:** Shih-Chi Hsu, Joshua K. Endow, Nicholas J. Ruppel, Rebecca L. Roston, Amy J. Baldwin, Kentaro Inoue

**Affiliations:** Department of Plant Sciences, University of California Davis, Davis, California, United States of America; University of South Florida College of Medicine, United States of America

## Abstract

Thylakoidal processing peptidase (TPP) is responsible for removing amino-terminal thylakoid-transfer signals from several proteins in the thylakoid lumen. Three TPP isoforms are encoded by the nuclear genome of *Arabidopsis thaliana*. Previous studies showed that one of them termed plastidic type I signal peptidase 1 (Plsp1) was necessary for processing three thylakoidal proteins and one protein in the chloroplast envelope *in vivo*. The lack of Plsp1 resulted in seedling lethality, apparently due to disruption of proper thylakoid development. The physiological roles of the other two TPP homologs remain unknown. Here we show that the three *A. thaliana* TPP isoforms evolved to acquire diverse functions. Phylogenetic analysis revealed that TPP may have originated before the endosymbiotic event, and that there are two groups of TPP in seed plants: one includes Plsp1 and another comprises the other two *A. thaliana* TPP homologs, which are named as Plsp2A and Plsp2B in this study. The duplication leading to the two groups predates the gymnosperm-angiosperm divergence, and the separation of Plsp2A and Plsp2B occurred after the Malvaceae-Brassicaceae diversification. Quantitative reverse transcription-PCR assay revealed that the two *PLSP2* genes were co-expressed in both photosynthetic tissues and roots, whereas the *PLSP1* transcript accumulated predominantly in photosynthetic tissues. Both *PLSP2* genes were expressed in the aerial parts of the *plsp1*-null mutant at levels comparable to those in wild-type plants. The seedling-lethal phenotype of the *plsp1*-null mutant could be rescued by a constitutive expression of Plsp1 cDNA but not by that of Plsp2A or Plsp2B. These results indicate that Plsp1 and Plsp2 evolved to function differently, and that neither of the Plsp2 isoforms is necessary for proper thylakoid development in photosynthetic tissues.

## Introduction

Oxygenic photosynthesis supports the lives of most organisms on earth. Capture of light energy and electron transport utilizing water as an electron donor occur in thylakoids, the internal membrane structures of cyanobacteria and chloroplasts in photosynthetic eukaryotes. As an endosymbiotic organelle, the chloroplast contains its own genome although most of its protein constituents are encoded in the nuclear genome [1]. Many of these nuclear-encoded proteins destined to chloroplasts are synthesized on cytosolic ribosomes as precursors with amino-terminal extensions called transit peptides. These precursor proteins first traverse the double-membrane envelope via the TOC and TIC (for translocon at the outer- and inner-envelope-membrane of chloroplasts) complexes [2]–[4]. Transit peptides are essential for proper protein targeting to the stroma and are removed by a soluble metallopeptidase [5]. Four distinct pathways have been identified to target proteins from the stroma to thylakoids: the cpSec (for chloroplast Sec) and cpTat (for chloroplast twin-arginine translocation) pathways direct proteins to the thylakoid lumen, whereas the cpSRP (for chloroplast signal recognition particle) and non-assisted spontaneous pathways catalyze targeting of thylakoid membrane proteins [6], [7]. All known nuclear-encoded cpSec and cpTat substrates, as well as some proteins that use the spontaneous pathway, carry bipartite transit peptides, which consist of thylakoid-transfer signals following the stroma-targeting transit peptides in their amino termini. The proteins with bipartite transit peptides include plastocyanins, the 33-, 23-, and 17-kD subunits of the oxygen-evolving-complex (OEC; PsbO, PsbP and PsbQ, respectively), some other photosystem components, lumen-located proteases and several other enzymes [8], [9]. The thylakoid-transfer signal is also found in chloroplast-encoded cytochrome f, which is sorted to the thylakoid membrane by the cpSec pathway [10]–[12]. During or soon after the translocation, the thylakoid-transfer signals are removed by thylakoidal processing peptidase (TPP) in the lumen.

Biochemical properties of TPP activity were extensively studied in the mid-1980's to the early 1990's [13]–[20]. The presence of TPP activity in complex chloroplasts of a heterokont alga was also reported [21]. The results revealed that TPP belongs to a group of membrane-bound serine proteases called the type I signal peptidase (SPase I) family. Members of the SPase I family are found in both prokaryotes and eukaryotes, cleaving intracellular or intraorganellar sorting signals in the amino termini of the translocated proteins [22]. In prokaryotes, SPases I are often called leader peptidases, which exist in the plasma membrane, apparently as monomeric forms, and remove the amino-terminal export signals from a number of proteins at the periplasmic space. Leader peptidases were shown to be essential for the viability of several Gram-negative and -positive bacteria [23]–[28]. In eukaryotes, there are two distinct SPase I activities in addition to TPP [22]. One is present in the endoplasmic reticulum as an oligomeric complex, cleaving the signal peptides either cotranslationally or posttranslationally [29]. Another activity is found in the mitochondria inner membrane. The enzyme responsible for this activity is called Imp (for inner membrane protease) and removes the intramitochondrial sorting signals from a subset of proteins in the space between the outer and inner membranes [30]. Imp was shown to comprise two subunits, each of which had catalytic activities with distinct specificities [31]. Biochemical studies revealed that SPases I recognize several short sequence motifs in the substrate proteins, notably small hydrophobic residues that are present at the −3 and −1 positions to the cleavage site. TPP showed more stringent requirements for these residues than other types of SPases I *in vitro* [20]. Beyond the conserved motifs, however, the substrate specificity of SPase I is relatively broad. For example, a bacterial SPase I could process thylakoid-transfer peptides whereas TPP could cleave bacterial export signals *in vitro* [18].

In 1998, the first TPP cDNA (At2g30440) was cloned from *Arabidopsis thaliana* based on its similarity to cyanobacterial SPases I in the coding sequence [32]. The carboxyl-terminal soluble domain of At2g30440 (residues 177–340) comprised catalytic residues conserved among SPases I. The antibody against this domain was shown to recognize a 30-kD protein in the thylakoid membrane. Furthermore, when produced in *E. coli*, this domain could process a cpTat substrate (the 23-kD subunit of OEC from wheat) although its activity was very low compared to the *E. coli* enzyme [30], [32]. Based on these results, At2g30440 was defined as the TPP although its *in vivo* function has not been demonstrated. Later, two additional TPP homologs (At1g06870 and At3g24590) were found to be encoded in the *A. thaliana* genome [30], [33]. A genetic study showed that one of them (At3g24590, which was termed as Plsp1 for plastidic SPase I 1) was required for proper chloroplast development [34]. *PLSP1* was originally found by screening for a gene encoding a protein responsible for complete maturation of Toc75, the protein translocation channel in the chloroplast outer envelope membrane. It turned out that the *plsp1*-null mutant accumulated unprocessed forms of not only Toc75 but also two cpSec substrates (PsbO and plastocyanin) and one cpTat substrate (PsbP) [34], [35]. The catalytic activity of Plsp1 could not be directly demonstrated *in vitro* [35]. Nonetheless, results of biochemical and electron microscopy-immunolocalization studies support the physical involvement of Plsp1 in protein maturation in both the envelope and thylakoids [36]. Inhibition of the complete maturation of Toc75 by the combination of site-directed mutagenesis and genetic complementation with the presence of Plsp1 did not affect proper chloroplast biogenesis [35]. Hence it was suggested that the accumulation of unprocessed lumenal proteins led to disruption of thylakoid development [35], [37]. These findings revealed the importance of protein maturation for thylakoid development. However, physiological roles of the other two TPP homologs in *A. thaliana* remain unknown.

In this work, we aimed to address the significance of gene duplications that gave rise to multiple TPP homologs in *A. thaliana*. We examined phylogenetic relationships of the TPP homologs, compared patterns of their gene expression, and used a genetic complementation assay with the seedling-lethal *plsp1*-null mutant to test their functional interchangeability. The obtained results revealed functional diversification of the TPP homologs.

## Results

### Gene duplication events that gave rise to multiple TPP homologs

**Figure 1:**
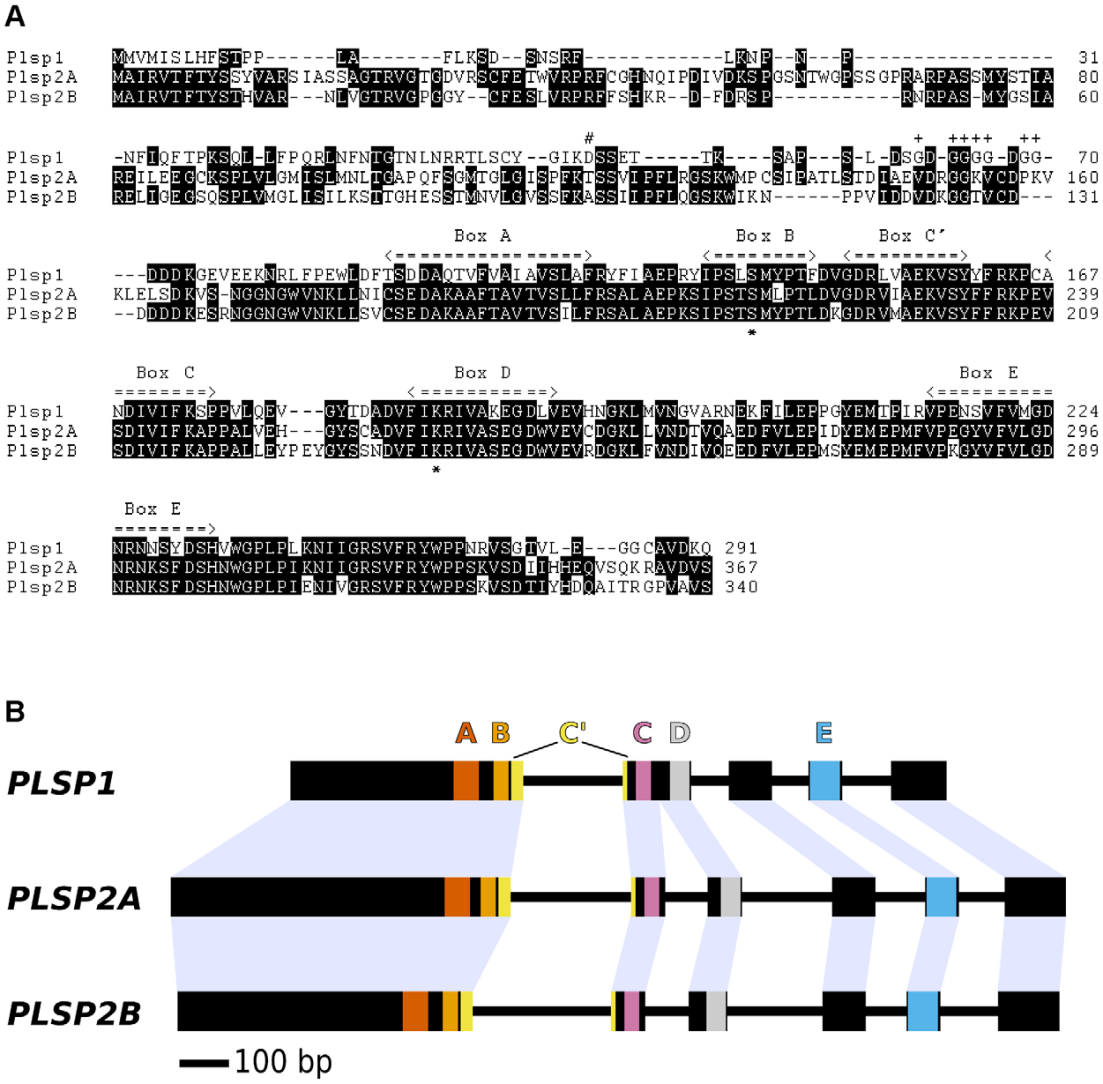
Three thylakoidal processing peptidase homologs in *Arabidopsis thaliana*. A) Alignment of predicted amino acid sequences of the three TPP homologs from *A. thaliana*. The numbers indicate amino acid residues for each protein. Residues conserved in at least two proteins are shown as white characters on a black background. The six sequence motifs conserved among SPases I (Boxes A–E and C′) [22], [38] are indicated. The conserved Ser and Lys residues shown to form the catalytic site are indicated with asterisks under the aligned sequences. The Asp residue predicted to be the first mature residue in Plsp1 by ChloroP [69], and Gly residues within the glycine-rich region of Plsp1 (see Figure S2) are indicated with a number sign (#) and plus sings (+), respectively. B) Comparison of structures of *A. thaliana TPP* genes. Exons and introns are indicated as boxes and solid lines, respectively. The regions of the genes coding for the conserved A, B, C′, C, D and E boxes are indicated by shading with the box. The coding sequence for box C′ is divided by the first intron in all three genes.

The three TPP homologs in *A. thaliana* are relatively diverse in their amino termini. However, At1g06870 has a higher overall sequence identity to At2g30440 (62%) than to Plsp1 (41%) (Figure 1A). In addition, as was reported [30], *AT1G06870* and *AT2G30440* genes are more similar to each other than they are to *PLSP1* in the exon-intron structure (Figure 1B). These data indicate that diversification of *PLSP1* occurred before that of the other two genes. Based on this, we named At1g06870 and At2g30440 as two Plsp2 isoforms, Plsp2A and Plsp2B, respectively.

**Figure 2:**
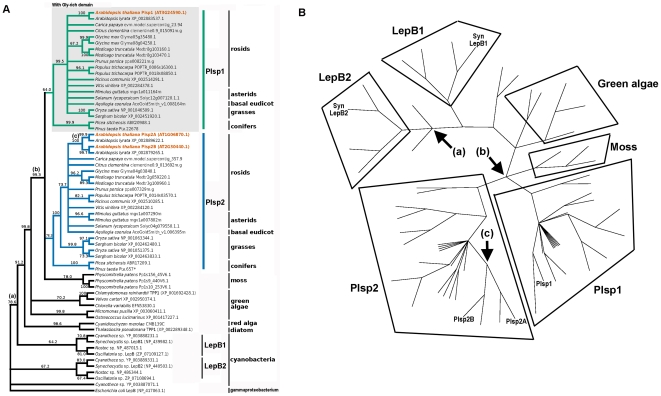
Phylogenetic tree for TPP-related sequences. A) A neighbor-joining tree was constructed using mean amino acid differences for highly conserved regions of predicted proteins with high similarity to Plsp1 from ten rosids [*Arabidopsis thaliana*, *Arabidopsis lyrata*, papaya (*Carica papaya*), clementine orange (*Citrus clementina*), soybean (*Glycine max*), *Medicago truncatula*, peach (*Prunus persica*), poplar (*Populus trichocarpa*), castorbean (*Ricinus communis*) and grape (*Vitis vinifera*)], two asterids [monkey flower (*Mimulus guttatus*) and tomato (*Solanum lycopersicum*)], one basal eudicot [columbine (*Aquilegia coerulea*)], two grasses [rice (*Oryza sativa*) and sorghum (*Sorghum bicolor*)], two conifers [spruce (*Picea sitchensis*) and pine (*Pinus taeda*)], a moss (*Physcomitrella patens*), five chlorophyte green algae (*Chlamydomonas reinhardtii*, *Volvox carteri*, *Chlorella variabilis*, *Micromonas pusilla*, and *Ostreococcus lucimarinus*), a red alga (*Cyanidioschyzon merolae*), a diatom (*Thalassiosira pseudonana*), four cyanobacteria (*Cyanothece* sp. PCC 7822, *Synechocystis* sp. PCC 6803, *Nostoc* sp. PCC 7120, and *Oscillatoria* sp. PCC 6506), and a gammaproteobacterium (*Escherichia coli*). For each protein, the name or predicted protein identifier for the respective database is given. Nodes present in more than 600 of the 1000 bootstrap trees are shown and the percentage value of trees supporting each node is indicated. In addition, the key branches [(a), (b), and (c)] described in the text are indicated. Proteins containing polyglycine stretches (Figure S2) are in the shaded box. *The Pta.657 sequence is incomplete so the presence of a polyglycine stretch is unknown. B) Another representation of the tree shown in A), emphasizing the relationships of TPP-related sequences from *E. coli*, cyanobacteria, and land plants.

To gain insights into the gene duplication events that gave rise to the three proteins in *A. thaliana*, we analyzed phylogenetic relationships between potential TPP homologs from a diverse set of land plants, green algae, a red alga, a diatom and cyanobacteria, with *Escherichia coli* SPase I as an outgroup. The tree was constructed based on the alignment of six common domains (A, B, C, C′, D and E) [22], [38] and their flanking regions that are conserved among SPases I (Figure S1). The resulting tree (Figure 2) provided three findings.

The first finding was that TPP may have originated before the endosymbiosis. A cyanobacterium *Synechocystis* sp. PCC6803 has two SPases I, LepB1 (sll0716) and LepB2 (slr1377). It was previously shown that LepB1 was predominantly involved in maturation of photosynthetic components, whereas LepB2 removed export signals from translocated proteins at the plasma membrane, similar to the indispensable SPases I in non-photosynthetic bacteria [28]. All four cyanobacteria examined in our study contained at least one each of the LepB1 and LepB2 homologs. LepB1 homologs were more similar to plant TPP homologs than to LepB2 homologs as shown by node (a) in Figure 2. These data suggest that the SPase I specific for photosynthetic components in the ancient cyanobacterium evolved to become TPP in photosynthetic eukaryotes, whereas the one in the plasma membrane for exported proteins was lost during the evolution of chloroplasts.

The second finding was the presence of two distinct groups of TPP in vascular plants: one includes Plsp1 and the other comprises the two Plsp2 isoforms from *A. thaliana* as shown by node (b) in Figure 2. All seed plants analyzed were found to contain at least one member in each of the Plsp1 and Plsp2 groups. This suggests that the two groups may have evolved to play diverse roles. In addition, every member in the Plsp1 family contained a unique glycine-rich domain between the predicted transit peptide and the transmembrane domain which was not found in Plsp2 orthologs (Figures 2A and S2). The data clearly indicate that the duplication that led to Plsp1 and Plsp2 predates separation of gymnosperms and angiosperms. However, our analysis did not address whether this duplication occurred prior to or after the diversification of vascular and non-vascular plants. This is because the three TPP homologs in the moss *Physicomitrella patens* formed their own clade, and the relationship of this clade to the Plsp1 and Plsp2 groups could not be resolved (node (b) in Figure 2).

Finally, the tree implies a relatively recent duplication that gave rise to Plsp2A and Plsp2B as shown by node (c) in Figure 2. This is consistent with their origin resulting from the whole-genome duplication event that appears to have occurred early in the evolution of Brassicaceae after their divergence from Malvaceae [39]. In fact, the *PLSP2A* and *PLSP2B* genes were found in a pair of duplicated regions on chromosomes 1 and 2 (Figure S3) [40]. Interestingly, each of the duplicated segments encodes paralogs of two other chloroplast proteins, the FtsH protease and the 23-kD subunit of OEC, PsbP; *PLSP2A* coexists with *FTSH8* and *PSBP1*, whereas *PLSP2B* occurs in the same segment as *FTSH2 (VAR2*) and *PSBP2* (Figure S3). Previous genetic studies showed that the functions of FtsH2 and FtsH8 are partially redundant: knockout of *FTSH2* resulted in a variegated phenotype, which could be rescued by overexpression of *FTSH8* [41] although the *fths8*-null mutant was indistinguishable from wild type [42]. By contrast, *PSBP1* may be the only gene encoding the functional 23-kD subunit of OEC because *PSBP2* appeared to be silenced [43]. Together, these data suggest that the two *PLSP2* genes may encode proteins with redundant functions, or that one of them may not be expressed.

### Distinct expression patterns of the *PLSP1* and *PLSP2* genes

Results of our phylogenetic analysis suggest that Plsp1 and Plsp2 may play distinct roles, and that Plsp2A and Plsp2B may be functionally redundant or one of them may not be functional. To test these possibilities, we examined expression patterns of the *TPP* genes in *A. thaliana*. First, we analyzed publicly available datasets by using the Genevestigator [44]. The existing ATH1 platform with useful datasets comprised oligonucleotide probes for *PLSP1* and *PLSP2A*, but not the one for *PLSP2B*. However, the *PLSP2A* probe may cross-react with the *PLSP2B* transcript as it showed a significant identity to part of the Plsp2B cDNA sequence (Figure S4A). When the data were plotted according to developmental stages, both *PLSP1* and *PLSP2A* genes were found to be expressed throughout the plant's life cycle, although their expression patterns were different (Figure S4B). *PLSP1* expression was relatively high from the germinated seed stage and peaked at bolting and young flower stages. By contrast, *PLSP2A* expression was relatively low in the germinated seed, bolting, and mature silique stages, and it peaked at the young rosettes and young flower stages. The difference between the two genes' expression patterns was also clear when the data were analyzed based on different tissues (Figure S4C). *PLSP1* expression was relatively high in embryos and photosynthetic tissues including cotyledons and leaf primordia, but was low in roots and hypocotyls. *PLSP2A* expression was, by contrast, high not only in photosynthetic tissues but also in roots, and was low in embryos.

**Figure 3:**
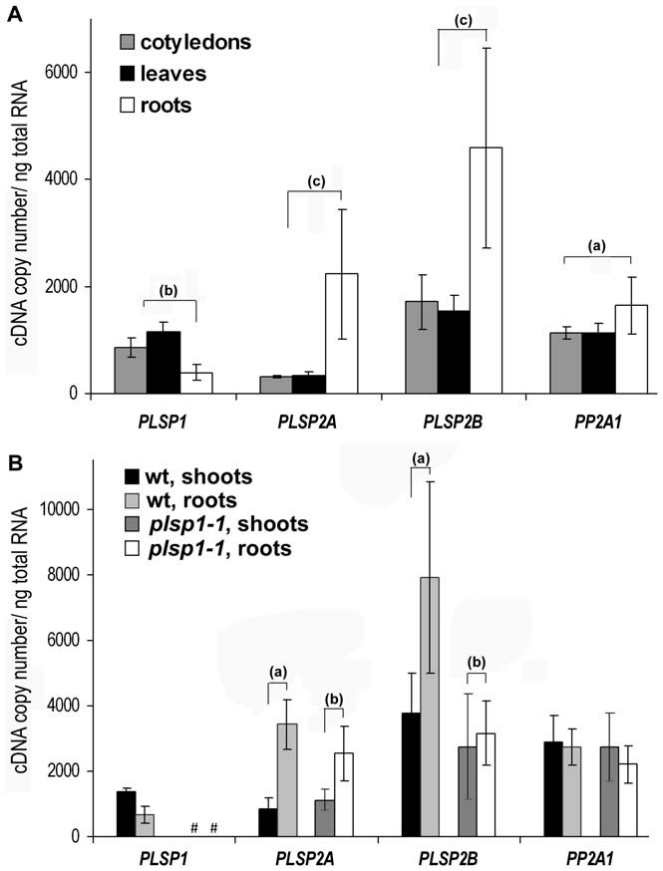
Expression of *TPP* genes. A) Expression of *PLSP1*, *PLSP2A*, *PLSP2B*, and *PP2A1* genes in wild-type *A. thaliana* seedlings by qRT-PCR. Cotyledons, leaves, and roots were collected from plants grown on MS media with 1% sucrose for 12 days. Data represent the mean of three independent biological replicates except those of wild-type roots which contain six repeats, and error bars indicate standard deviations. (a)–(c) indicate comparisons of expression levels between different parts of plants. B) Expression of *PLSP1*, *PLSP2A*, *PLSP2B*, and *PP2A1* genes in wild type and mutant (*plsp1-1*) *A. thaliana* seedlings by qRT-PCR. Shoot and root samples were collected from plants grown on MS media with 3% sucrose for 12 days. Data represent the mean values and standard deviations of three biological repeats. # indicates the values were below detection limit (25-copy number of standards in a 25 µl reaction volume). (a) and (b) indicate comparisons of expression levels between different parts of plants.

The *in silico* data support the idea that Plsp1 and Plsp2 may have diverse functions, but do not address whether the two *PLSP2* genes were co-expressed, and if either of them was silenced. Hence, we used quantitative reverse transcription (qRT)-PCR to estimate the level of transcripts encoding the three TPP homologs in leaves, cotyledons, and roots from plate-grown *A. thaliana* seedlings. In order to increase the accuracy of gene expression profiling, we included a reference gene, *PP2A1* (*AT1G59830*), which encodes a catalytic subunit of Ser/Thr protein phosphatase 2A and whose expression pattern appeared to be consistent over a wide range of developmental stages by microarray analyses [45]. Our results showed that indeed the expression levels of *PP2A1* in the three organs were comparable (P>0.1; Figure 3A (a)). By contrast, consistent with the *in silico* data, *PLSP1* expression was higher in the aboveground photosynthetic organs (leaves and cotyledons) than in roots (Figure 3A (b)): when normalized with the *PP2A1* transcript level (see Materials and Methods), *PLSP1* expression was found to be 2.9-fold higher in leaves than in roots (P<0.01). Interestingly, on the contrary, the expression of both *PLSP2* genes was higher in roots than in photosynthetic organs (Figure 3A (c)): when the *PP2A1* transcript level was used for normalization, *PLSP2A* and *PLSP2B* transcript levels were found to be 4.5- and 2-fold higher in roots than in leaves (P<0.01), respectively. The quantitative analysis also revealed that in leaves, the level of the *PLSP1* transcript was 3.5 times higher than that of the *PLSP2A* transcript (P<0.01), but was comparable with that of *PLSP2B* (P>0.1). In roots, by contrast, *PLSP2A* and *PLSP2B* transcripts were 5.65 and 11.6 times more abundant than the *PLSP1* transcript (P<0.05). Together, the results showed that both *PLSP2A* and *PLSP2B* genes were expressed in a similar pattern. The qRT-PCR data also confirmed the distinct expression profiles of *PLSP1* and the two *PLSP2* genes.

Suppressing gene expression of one isoform often results in enhanced gene expression of another isoform as a mechanism of compensation, such as enhanced expression of *PSBO2* in the *psbo1*-null mutant [46], [47]. To test if this was also the case for TPP isoforms, we examined expression of the *PLSP2A* and *PLSP2B* genes in the *plsp1*-null mutant. As previously reported, this mutant was seedling-lethal and its development required supplementation of the media with 3% sucrose [35]. Even in this condition, it was technically difficult to separate leaves and cotyledons from other parts of the mutant plants. Hence we combined the aboveground parts together and used them as the source of RNA for the analysis. As shown in Figure 3B, disruption of *PLSP1* expression did not lead to increased expression of either of the *PLSP2* genes. Instead, expression the *PLSP2* genes in roots was reduced in the *plsp1*-null mutant. This was clearer if the ratios of the expression in the roots and shoots were considered (Figure 3B, compare (a) and (b)): when normalized with the *PP2A1* transcript level, the ratios of the transcript level in roots to that in shoots of wild type were 4.3∶1 for *PLSP2A* and 2.2∶1 for *PLSP2B* (P<0.01), whereas those in the mutant were 2.2∶1 for *PLSP2A* (P<0.01) and 1.5∶1 for *PLSP2B* (P<0.05). These data may indicate that the lack of Plsp1 affected proper plastid development in roots, generating a retrograde signal to suppress expression of a subset of nuclear genes including *PLSP2*.

### Accumulation of TPP proteins in chloroplasts

**Figure 4:**
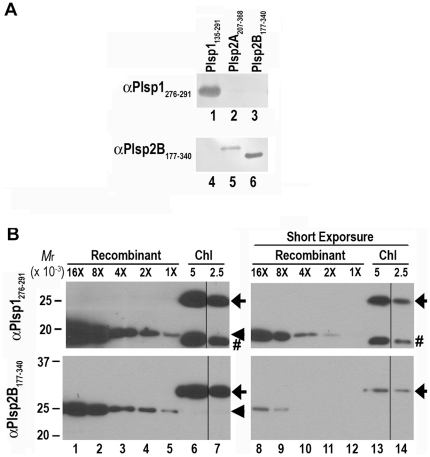
The presence of TPP proteins in *A. thaliana*. A) Immunoreactivity of the antisera against TPP homologs. Bacterially-produced TPP homologs (100 ng each of residues 135–291 of Plsp1, 207–368 of Plsp2A, and 177–340 of Plsp2B) were separated by 12% SDS-PAGE and analyzed by immunoblotting using the antisera indicated at left. B) Estimation of the level of TPP homologs in *A. thaliana* chloroplasts. Varying amount of bacterially produced recombinant proteins [Recombinant; Plsp1_135–291_ (top) and Plsp2B_177–340_ (bottom); 15 ng (16×) to 0.94 ng (1×)] and chloroplasts from wild type containing 5 and 2.5 µg chlorophylls were separated by SDS-PAGE and analyzed by immunoblotting with antibodies indicated at left. Signals were visualized by an enhance chemiluminescence method. To better estimate the amount of the TPP proteins in chloroplasts, images of a shorter exposure were also presented at right. The recombinant proteins are indicated with arrowheads, whereas the proteins in the chloroplasts are indicated with arrows. Because the recombinant proteins lacked transmembrane domains of the mature forms, their mobility did not correspond to that of the endogenous proteins. The number signs indicate the non-specific protein recognized by the αPlsp1_276–291_ antibody.

To complement the gene expression analysis, we wished to examine the level of TPP proteins. Prior to the present study, two antisera against TPP homologs were available. The first antibody, which was against residues 177–340 of Plsp2B (Plsp2B_177–340_), was shown to recognize a protein of 30 kD in *A. thaliana* thylakoids [32]. The second antibody was raised against the unique carboxyl-terminal sequence of Plsp1 (residues 276–291; Plsp1_276–291_), and was shown to recognize a protein of 25 kD, which existed predominantly in thylakoids of mature chloroplasts [36]. Because of the similarities between the three TPP homologs, in particular the high sequence identity between Plsp2A and Plsp2B (Figure 1A), we wished to evaluate the specificity of these antisera. To this end, we examined their cross-reactivity with recombinant forms of the three TPP homologs, which were produced in *E. coli*. As expected, the αPlsp1_276–291_ antibody reacted specifically with Plsp1 (Figure 4A, compare lanes 1–3). By contrast, the αPlsp2B_177–340_ antibody recognized not only Plsp2B, but also Plsp2A (Figure 4A, lanes 5 and 6), but not Plsp1 (Figure 4A, lane 4). The cross-reactivity of the αPlsp2B_177–340_ antibody with Plsp2A is likely due to the high sequence identify (82%) between the two Plsp2 isoforms within the region used as an antigen. The antisera against the unique carboxyl termini of the two Plsp2 isoforms did not appear to recognize the endogenous proteins (Shih-Chi Hsu, Rebecca Roston, and Kentaro Inoue, unpublished). Hence we used the αPlsp2B_177–340_ antibody to compare the amounts of Plsp1 and Plsp2 isoforms in chloroplasts isolated from *A. thaliana* seedlings. Based on the immunoblotting data (Figure 4B), the levels of Plsp1 and Plsp2 (equivalent to Plsp2B) proteins were calculated to be approximately 0.15 ng and 0.3 ng per µg chlorophyll, respectively. These data corresponded well with the qRT-PCR data, showing that the total amount of *PLSP2A* and *PLSP2B* transcripts was slightly larger than the amount of *PLSP1* transcripts in leaves (Figure 3A).

### Functional interchangeability of TPP homologs using the seedling lethal *plsp1*-null mutant

**Figure 5:**
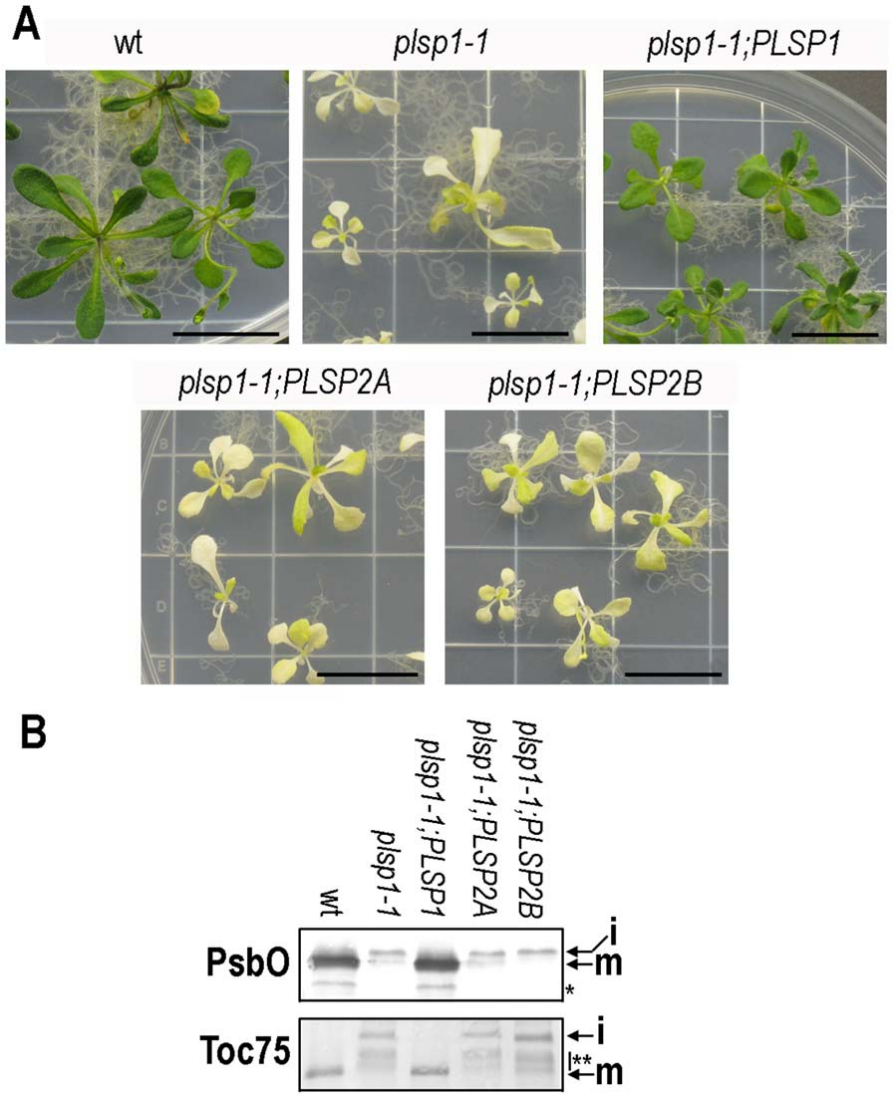
Genetic complementation assay. A) *A. thaliana* seedlings grown for 21 days used for the analyses. Scale bars indicate 20 mm. B) Total proteins (25 µg protein) extracted from wild type (wt) and the mutant *A. thaliana* seedlings were separated by 15% (for PsbO) or 7.5% (for Toc75) SDS-PAGE and analyzed by immunoblotting with antisera against proteins shown at left. i and m indicate unprocessed intermediate and mature forms, respectively. An asterisk indicates the unidentified bands that ran faster than mature form of PsbO. A double asterisk indicates bands that are larger than the properly processed form of Toc75 as shown before [35].

Disruption of the *PLSP1* gene resulted in seedling lethality and accumulation of a subset of unprocessed proteins in the envelope and thylakoids [34], [35]. The available data suggest that the endogenous level of *PLSP2A* and *PLSP2B* gene expression could not overcome the lack of the functional Plsp1 protein. We wished to test if this was due to the functional difference between Plsp1 and Plsp2 isoforms, or insufficient expression of the *PLSP2* genes, although the level of their transcripts appeared to be significantly high in the *plsp1*-null mutant (Figures 3B). To this end, we decided to make use of the genetic complementation system that was used to confirm that the seedling lethal phenotype of the mutant was due to the knockout of *PLSP1* [34]. As shown in Table 1 and Figure 5, the construct carrying the Plsp1 coding sequence successfully complemented the mutant phenotype. By contrast, all the *plsp1*-null plants carrying a coding sequence for either Plsp2A or Plsp2B were indistinguishable from non-transformed *plsp1*-null plants in their properties, including visible phenotype (Figure 5A) and the size of PsbO and Toc75 (Figure 5B). Comparative RT-PCR clearly demonstrated the expression of the inserted transgene encoding Plsp2 proteins in these plants (Figure S5C). These results support the idea that Plsp2A and Plsp2B were functionally distinct from Plsp1.

**Table 1:** Segregation of *plsp1-1* plants transformed with TPP-encoding sequences.

Proteins encoded by transgene	Generation	# of seeds sowed	# of plants selected^c^			Genotype^d^	
			green	white	g ∶ w ratio	Green	White
						+/+; +/−; −/−	+/+; +/−; −/−
Plsp1	T1	∼2800	82	0	–	8; 21; 6	–
	T2^a^	∼2800	27	0	–	0; 0; 27	–
Plsp2A	T1	∼2800	52	26	2 ∶ 1	7; 20; 0	0; 0; 10
	T2^b^	∼1400	45	16	2.8 ∶ 1	7; 3; 0	0; 0; 16
Plsp2B	T1	∼9100	223	104	2.1 ∶ 1	6; 18; 0	0; 0; 7
	T2^b^	∼1400	38	13	2.9 ∶ 1	5; 5; 0	0 ;0; 13

a Segregation from two independent viable lines confirmed to be homozygous for *plsp1-1* was analyzed.

b Segregation from a viable line confirmed to be heterozygous for *plsp1-1* was analyzed.

c Plants that developed true leaves after 7-day incubation on MS media containing 3% sucrose and 20 µg/ml hygromycin at 24°C, 19 h/day of light (∼100 µmole m^−2^ s^−1^), were defined as “selected”. “Green” and “White” seedlings were visually identified. T1 plants that were selected may have included non-transformed plants susceptible to hygromycin.

d Genotypes of a subset of selected plants were analyzed by genomic PCR.

## Discussion

Proper targeting of many photosynthetic proteins and enzymes located in the thylakoid lumen requires amino-terminal thylakoid-transfer signals, which are removed by TPP upon translocation. The catalytic properties of TPP activity were defined by extensive biochemical studies in the 1980's to 1990's. By the time the first TPP cDNA was cloned from *A. thaliana* [32], however, research on this enzyme appeared to have diminished, if not completely disappeared. Although two additional TPP homologs were identified in *A. thaliana* [30], no work had been reported to elucidate their physiological roles until a recent genetic study demonstrating that one of them (Plsp1) was involved in proper thylakoid development [34], [35]. The aim of the present work was to elucidate the nature and significance of multiple TPP isoforms.

Our phylogenetic data suggest that TPP originated from the duplication of a SPase I in an ancient cyanobacterium. Sometime between the emergence of land plants and the separation of gymnosperms and angiosperms, TPP evolved further into two groups. The first group includes Plsp1, which is responsible for maturation of PsbO, PsbP, plastocyanin in thylakoids and Toc75 in the envelope membrane [34], [35]. The second group comprises Plsp2 isoforms including Plsp2B, the first TPP whose cDNA was cloned from any eukaryotes [32]. The conservation of the glycine-rich domain in the Plsp1 orthologs but not in other TPP-related proteins (Figure S2) implies that this feature was acquired after the divergence of Plsp1 and Plsp2 paralogs. It remains unknown whether this glycine-rich domain plays a role in targeting of Plsp1 to the chloroplast envelope as in the case of the polyglycine stretch of Toc75 [48], [49]. Furthermore, the gene expression profile and the mobility on BN-PAGE suggest that Plsp1 and Plsp2 isoforms may function differently. This idea was further corroborated by the results of the assays using the *plsp1*-null mutant showing that neither of the Plsp2 isoforms could substitute for Plsp1 in its function. Although many questions remain to be addressed, the data presented in this study clearly indicated that the gene duplication that gave rise to the two TPP groups caused functional diversification.

The available data suggest that Plsp1 functions predominantly in chloroplasts although it should exist in non-photosynthetic plastids, too, to process the general protein translocation channel Toc75 in the envelope membrane [34]. The results obtained in this study also suggest that Plsp2 isoforms' function may be more prominent in roots than in leaves, although their accumulation in chloroplasts was significant. Because their genes are co-expressed, the two Plsp2 isoforms may function redundantly and/or exist in the same oligomeric complexes, similar to the case of the two Imp isoforms in mitochondria [30], [31]. Do Plsp2 isoforms function as a TPP in chloroplasts? If so, what are their substrates? Chaal et al. showed a low processing activity of the bacterially-produced catalytic site of Plsp2B against wheat PsbP *in vitro* [32]. However, the *plsp1*-null plant, which contained a significant amount of the Plsp2 protein (Figure 4D), accumulated the unprocessed form of PsbP [35]. One possibility is that Plsp2B requires the presence of Plsp1 to properly process PsbP, although it is also possible that PsbP is not the substrate of Plsp2B *in vivo*. Interestingly, among TPP substrates examined, the 17-kD subunit of OEC (PsbQ) and FtsH2/8 appeared to accumulate as mature forms in the *plsp1*-null mutant [35]. Therefore Plsp2 isoforms may be responsible for processing of these thylakoidal proteins instead of PsbP. Another potential Plsp2 substrate is an inner envelope protein, Tic40, as suggested by Firlej-Kwoka et al. [50]. Tic40 carries a bipartite transit peptide and was shown to be processed to its mature size by the *E. coli* SPase I *in vitro* [51]. Indeed, the sizes of Tic40 were indistinguishable between the *plsp1*-null mutant and wild type [34], [35]. Hence, similar to the case of Plsp1, Plsp2 isoforms may play roles in processing of proteins in both the envelope and thylakoids. It is noteworthy to mention that genetic data suggest that LepB1 of *Synechocystis* sp. PCC6803 may exist in both the thylakoid and plasma membranes of the bacterium [28]. Hence, dual localization of TPP may have originated in the ancient cyanobacterium.

What is the function of Plsp2 isoforms in root plastids, which appear to have very limited thylakoid network [52]? A recent study identified the presence of cpSec translocon homologs in root plastids, most probably in their envelope membrane [53]. Similarly, Plsp2 may be located in the envelope membrane of root plastids. Analysis of publicly available microarray databases revealed that expression of genes encoding most of known and putative TPP substrates including Tic40 was relatively low in roots (Kentaro Inoue, unpublished). However, genes for several TPP substrates including FtsH2 and FtsH5, which were recently demonstrated to utilize distinct sorting pathways [54], were found to be expressed at a significant level in roots (Kentaro Inoue, unpublished). We will need to establish a system to determine whether these proteins are located in the scarce thylakoid network or in the envelope membrane within root plastids.

We identified a *plsp2b*-null mutant (SALK_000738), which was indistinguishable from wild type in its growth phenotypes (Yi-Tze Chen and Kentaro Inoue, unpublished). Interestingly, chloroplasts isolated from this mutant accumulated the protein recognized by the αPlsp2B_177–340_ antibody at a level comparable to that in wild type, although the presence of the *PLSP2B* transcript was under the detection limit (Shih-Chi Hsu, Nicholas Ruppel, Robert Shih and Kentaro Inoue, unpublished). This result may indicate that the immunoreactive protein was derived from the *PLSP2A* gene, and may also support the idea that the two Plsp2 isoforms were functionally redundant. Future research including generation and analysis of the mutant plant that lacks both Plsp2 isoforms should allow us to develop a specific hypothesis about the functions of Plsp2. Furthermore, defining the localization and physiological roles of TPP isoforms in roots should help us understand the properties and functions of root plastids, which have been under-appreciated in the field of organelle biology.

## Materials and Methods

### Phylogenetic analysis

Protein sequences with high similarity (Expect value less than 1×e^−20^ except for *Escherichia coli* LepB which was used despite having a higher E-value of 2×e^−10^) to Plsp1 (AT3G24590.1) were identified by a blastp search against The Arabidopsis Information Resource protein database for *Arabidopsis thaliana* Columbia (http://www.arabidopsis.org), the National Center for Biotechnology Information non-redundant protein sequences for *Arabidopsis lyrata* subsp. *lyrata*, *Ricinus communis* Hale, *Vitis vinifera* PN40024, *Oryza sativa* Japonica Nipponbare, *Sorghum bicolor* BTx623, *Picea sitchensis*, *Chlamydomonas reinhardtii* CC-503 cw92 mt+, *Volvox carteri* f. *nagariensis* Eve, *Chlorella variabilis* NC64A, *Micromonas pusilla* CCMP1545, *Ostreococcus lucimarinus* CCE9901, *Thalassiosira pseudonana* CCMP1335, *Nostoc* sp. PCC 7120, *Cyanothece* sp. PCC 7822, *Oscillatoria* sp. PCC 6506, *Synechocystis* sp. PCC 6803 and *Escherichia coli* K-12 MG1655 (http://www.ncbi.nlm.nih.gov), *Cyanidioschyzon merolae* Genome Project annotated CDS for *Cyanidioschyzon merolae* 10D [55], SOL Genomics Network ITAG Release 2 predicted proteins database for *Solanum lycopersicum* [56], Mt3.5 genome assembly release International Medicago Genome Annotation Group protein database for *Medicago truncatula* (http://www.medicagohapmap.org), and Phytozome release v6.0 (http://www.phytozome.net) predicted protein databases for *Carica papaya* [57], *Citrus clementina* (Haploid Clementine Genome, International Citrus Genome Consortium, 2011, http://int-citrusgenomics.org/, http:://www.phytozome.net/clementine), *Glycine max* [58], *Populus trichocarpa* [59], *Mimulus guttatus* (*Mimulus* Genome Project, DoE Joint Genome Institute), *Prunus persica* (International Peach Genome Initiative), *Aquilegia coerulea* Goldsmith (*Aquilegia* Genome Sequencing Project, DoE Joint Genome Institute) and *Physcomitrella patens* subsp. *patens* Gransden 2004 [60]. The NCBI UniGenes Pta.22678 and Pta.657 from *Pinus taeda* were also used for analysis. Sequences of putative mitochondrial Imp proteins were identified by blastp against the NCBI non-redundant protein sequences database, and were not included in further analysis. Amino acid sequences were aligned using the accurate mode of T-COFFEE version 8.99 [61] and minor adjustments were made manually (Figure S1). Sequences containing the conserved Boxes A, B, C′, C, D and E [22], [38], which correspond to the residues 101–162, 164–179 and 184–258 of Plsp1, were used for phylogenetic analysis. To generate Figure S2, greater than 85 amino acid residues amino terminal of Box A of land plant sequences, except for the *Pinus taeda* UniGenes which were incomplete, were added. The analysis was performed using the PHYLIP phylogeny package version 3.69 [62]. Mean character distances for 1000 bootstrap datasets were calculated using the Jones-Taylor-Thornton matrix model in PROTDIST. These were then used to build a tree using neighbor-joining in NEIGHBOR. A consensus tree displaying nodes supported by at least 600 bootstrap trees (60%) was produced.

### Cloning of cDNA sequences encoding *A. thaliana* TPP homologs

Subcloning of coding sequence for Plsp1 into the pGEM®-T Easy vector (Promega, Madison, WI) was described previously [34]. The Plsp2A-coding sequence was amplified from 13-day-old *A. thaliana* seedlings by PCR using a set of primers shown in Table S1 and subcloned into the pGEM®-T Easy vector. Plsp2B cDNA was obtained from the Arabidopsis Biological Resource Center (The Ohio State University, Columbus, OH) in the pUNI51 vector as clone U69173.

For production of the catalytic domains of TPP homologs in *E. coli*, the construct encoding residues 177–340 of Plsp2B in vector pET16b-Kan termed pET-TPP558 [32] was a kind gift of Dr. Christopher J. Howe (University of Cambridge). cDNA sequences encoding residues 135–291 of Plsp1 and 207–368 of Plsp2A were amplified by using primers listed in Table S1 and the plasmids described above as templates, and subcloned into the pET16b-kan vector using the *Bam*HI site. The resultant plasmids as well as pET-TPP558 were individually transformed into BL21(DE3)pLysS cells (Invitrogen, Carlsbad, CA), and proteins recovered in the inclusion bodies were purified by using Ni-NTA column following the manufacturer's instruction (Qiagen, Valencia, CA).

### Plant materials, growth conditions, and qRT-PCR

Seeds of wild-type and mutant *A. thaliana* (Columbia-0) were sown on Gel Drying Film (Promega) placed on top of Murashige-Skoog (MS) media containing Gamborg's vitamins (Caisson Laboratories, North Logan, UT) and 1% (for the assay in Figure 3A) or 3% sucrose (for the assay in Figure 3B) and solidified with 0.7% Phytoagar (Invitrogen) in square plates. After stratification in dark at 4°C for 3 days, plates were transferred to 24°C with 19 hour light per day and incubated for 12 more days. Cotyledons, the first pairs of true leaves, and roots were then separated, frozen in liquid nitrogen, and stored at −80°C before the analysis. RNA was extracted from the stored tissues using RNeasy Plant Mini Kit (Qiagen), quantified spectrophotometrically at 260 nm, and an aliquot of the samples (1.46 µg and 0.9 µg RNA for plants grown on 1% and 3% sucrose media, respectively) was reverse-transcribed using SuperScript™III with random primers (Invitrogen). The resultant cDNA was used as a template for PCR performed in 7300 Real-Time PCR System (Applied Biosystems, Foster City, CA) with FastStart Universal SYBR Green Master Mix (Roche, Indianapolis, IN) for quantification of PCR products and gene specific primers (Table S1), which were designed using Primer Express v2.0 (Applied Biosystems). Absolute copy number of cDNA for each gene was calculated by Sequence Detection Software v1.2.3 (Applied Biosystems) using external standard curves generated with linearized plasmid DNA. For normalization, the fold difference between the calculated cDNA copy numbers of the TPP isoform gene to that of *PP2A1* for each biological replicate was calculated and used.

### Sources of antibodies and immunoblotting

Antibodies against residues 276–291 of Plsp1 and PsbO (the 33-kD subunit of OEC) were prepared as described [36]. The antibody against residues 177–340 of Plsp2B was raised in rabbits (Zymed Laboratories, San Francisco, CA), and was further purified by using 30 µg of the antigen protein coupled to 100 µL of UltraLink iodoacetyl gel (MicroLink peptide coupling kit; Pierce) in the provided minicolumn according to the manufacturer's instructions as described [63]. Immunoblotting assays were done as described [64]. Amounts of proteins analyzed were quantified by Bradford's method using BSA as standard [65].

### Chloroplast isolation

Chloroplasts were isolated from *A. thaliana* seedlings grown on MS media supplemented with 2% sucrose and 0.7% Phytagar at 24°C with 19 h light per day for 3 to 5 weeks by a grinding method as described [64], except that the grinding buffer was modified to 50 mM HEPES-KOH, 330 mM sorbitol, 2 mM EDTA, and 2% (w/v) BSA, pH 8.0.

### Preparation and analysis of *plsp1-*null mutants transformed with constructs encoding TPP homologs

In the previous work, we used the binary vector pBIG-HYG [66] to complement the *plsp1-*null mutant [34]. In this work, we used Gateway® technology to facilitate cloning. Briefly, each of the TPP coding sequences was amplified from the plasmid described above with iProof High-Fidelity DNA Polymerase (Bio-Rad, Hercules, CA) using gene specific primers and Gateway *attb1* adapter primers (Table S1). The PCR products were subcloned into pDONR™221 using the Gateway BP clonase (Invitrogen) to generate entry clones. After confirmation of the sequences, the cloned fragments were transferred to pMDC32 [67] by the Gateway LR clonase reaction (Invitrogen). The resultant plasmids were transformed into *plsp1-1* heterozygous plants via the *Agrobacterium*-mediated floral dip method [68] and putative transformants were selected on the half-strength MS media containing vitamins supplemented with 3% sucrose, 0.8% Phytoblend agar (Caisson Laboratories), and 20 µg per mL of hygromycin.

For genotyping, a subset of plants grown on the media containing hygromycin were transferred to a fresh MS media containing hygromycin and used as the source of genomic PCR with primers listed in Table S1. For RT-PCR assay, RNA was extracted from a pool of leaves from seedlings grown on plates for 21 days and determined to be isogenic. Extraction and quantification of RNA, and reverse transcription of a total of 1 µg RNA were done as described above for qRT-PCR. Primers used for PCR are listed in Table S1, except those for the internal control (QuantumRNA 18S Internal Standards; Ambion, Austin, TX). For immunoblotting assay, total protein was extracted by 0.1 M Tris-HCl pH 6.8, 1% SDS, 15% glycerol, 5% β-ME from approximately 100 mg of the same fresh samples used for RT-PCR assay as described above.

## Supporting Information

Figure S1 **Alignment of predicted amino acid sequences of TPP-related proteins used for the phylogenetic analysis.** The conserved segments designated as A, B, C′, C, D and E boxes (Carlos et al. 2000; Paetzel et al. 2002) are shown. The numbers indicate amino acid residue numbers of Plsp1.
(TIFF)

Figure S2 **The presence of glycine-rich domains in the Plsp1 orthologs.** The sequences of amino-terminal 70 amino acids flanking the conserved Box A are aligned. Numbers correspond to those of the Plsp1 sequence. A polygl stretch was defined as a stretch of ten amino acid residues containing at least six glycine residues.
(TIF)

Figure S3 **A pair of duplicated segments in chromosomes 1 and 2 of** ***A. thaliana*** **nuclear genome (Block 0102031203980) that include three genes** ***PLSP2*** **,** ***FTSH*** **, and** ***PSBP*** **.**
(TIF)

Figure S4 A) Alignment of the *PLSP2A* probe and the *PLSP2B* sequences. B) *In silico* data for the expression of *TPP* genes in *A. thaliana* according to the development stages of the plants. C) *In silico* data for the expression of *TPP* genes in *A. thaliana* according to different tissue types.
(TIF)

Figure S5 A) Genomic PCR of wild-type (wt) and mutant *A. thaliana* seedlings. E and I indicate reactions specific to amplify the inserted T-DNA into *PLSP1* (918 bp) and part of the endogenous *PLSP1* (536 bp). B) Genomic PCR of wild-type and mutant *A. thaliana* seedlings. Presented are reactions specific to amplify the transgene introduced into the *plsp1*-null mutant encoding Plsp1 (437 bp), Plsp2A (1070 bp), and Plsp2B (595 bp). Far right lanes show the reactions using the plasmid used for transformation. C) RT-PCR profiles of wild-type and mutant *A. thaliana* seedlings for genes indicated at left. Each reaction contained two sets of primers: one for each cDNA whose size is indicated at right, and another for cDNA derived from 18S RNA indicated with an asterisk. The template used was either total RNA without (−) or with reverse transcription (+, RT).
(TIF)

Table S1 **Sequences of oligonucleotide primers used in this study.** All sequences are depicted from 5′ to 3′. F and R indicate forward and reverse primers, respectively.
(DOC)
