# Supplementary figures and images for: Functional Diversification of Thylakoidal Processing Peptidases in Arabidopsis thaliana
Source: PLoS One. 2011 Nov 7;6(11):e27258. doi: 10.1371/journal.pone.0027258 (PMC3210150; doi:10.1371/journal.pone.0027258)

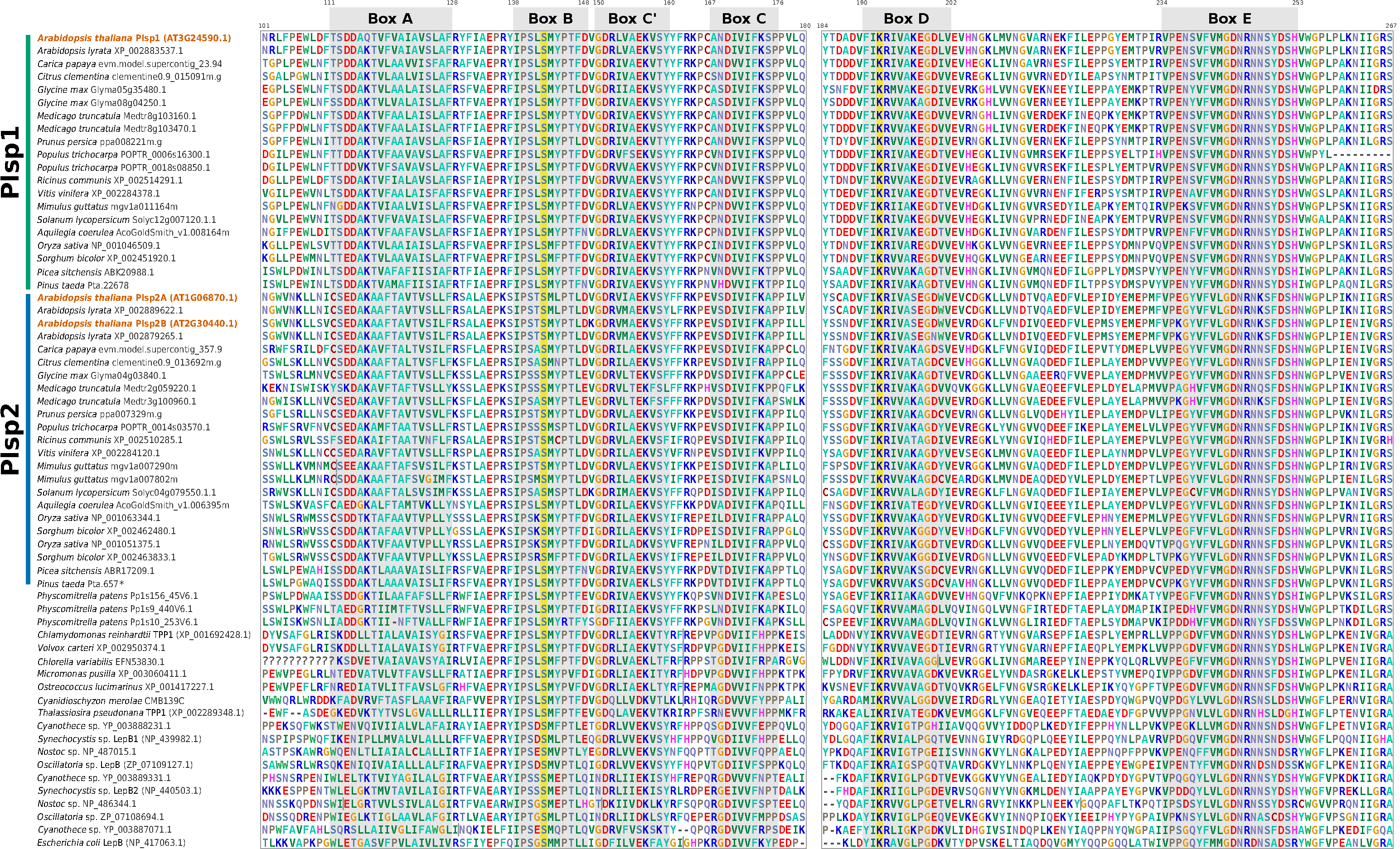

Supplement: Figure S1 — Alignment of predicted amino acid sequences of TPP-related proteins used for the phylogenetic analysis. The conserved segments designated as A, B, C′, C, D and E boxes (Carlos et al. 2000; Paetzel et al. 2002) are shown. The numbers indicate amino acid residue numbers of Plsp1. (TIFF) [file pone.0027258.s001.tiff]

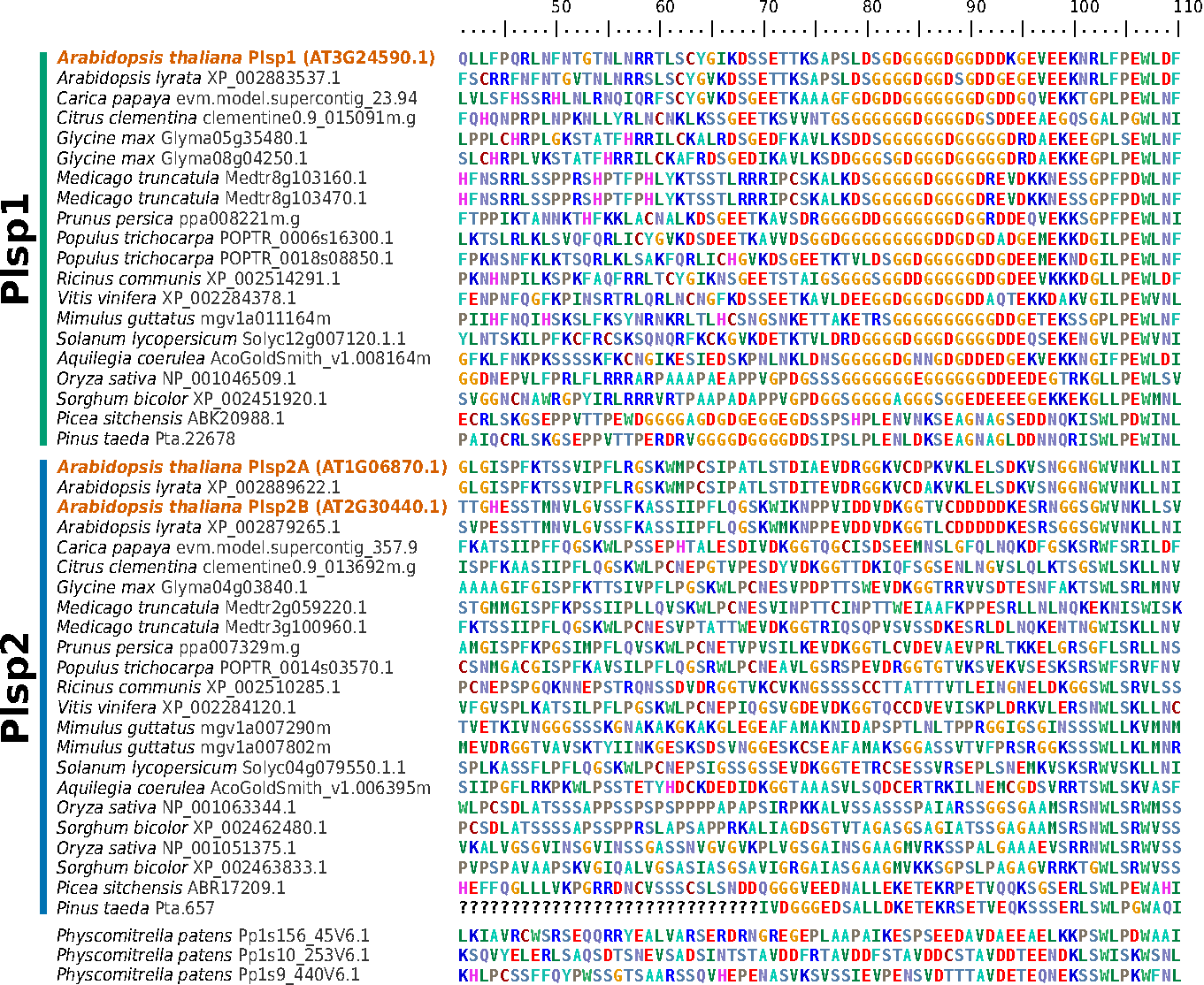

Supplement: Figure S2 — The presence of glycine-rich domains in the Plsp1 orthologs. The sequences of amino-terminal 70 amino acids flanking the conserved Box A are aligned. Numbers correspond to those of the Plsp1 sequence. A polygl stretch was defined as a stretch of ten amino acid residues containing at least six glycine residues. (TIF) [file pone.0027258.s002.tif]

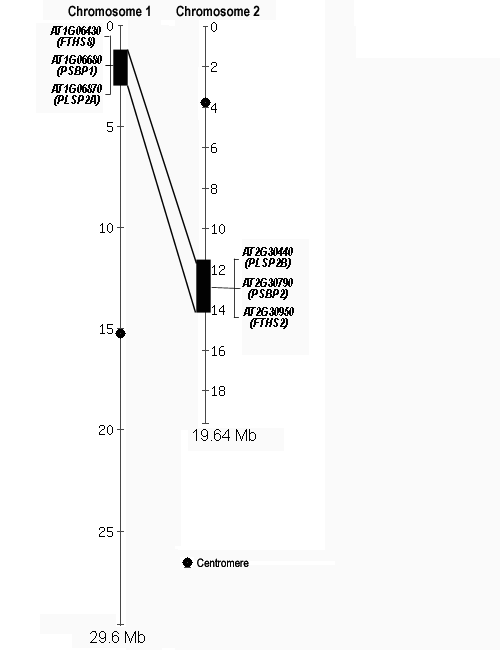

Supplement: Figure S3 — A pair of duplicated segments in chromosomes 1 and 2 of A. thaliana nuclear genome (Block 0102031203980) that include three genes PLSP2 , FTSH , and PSBP . (TIF) [file pone.0027258.s003.tif]

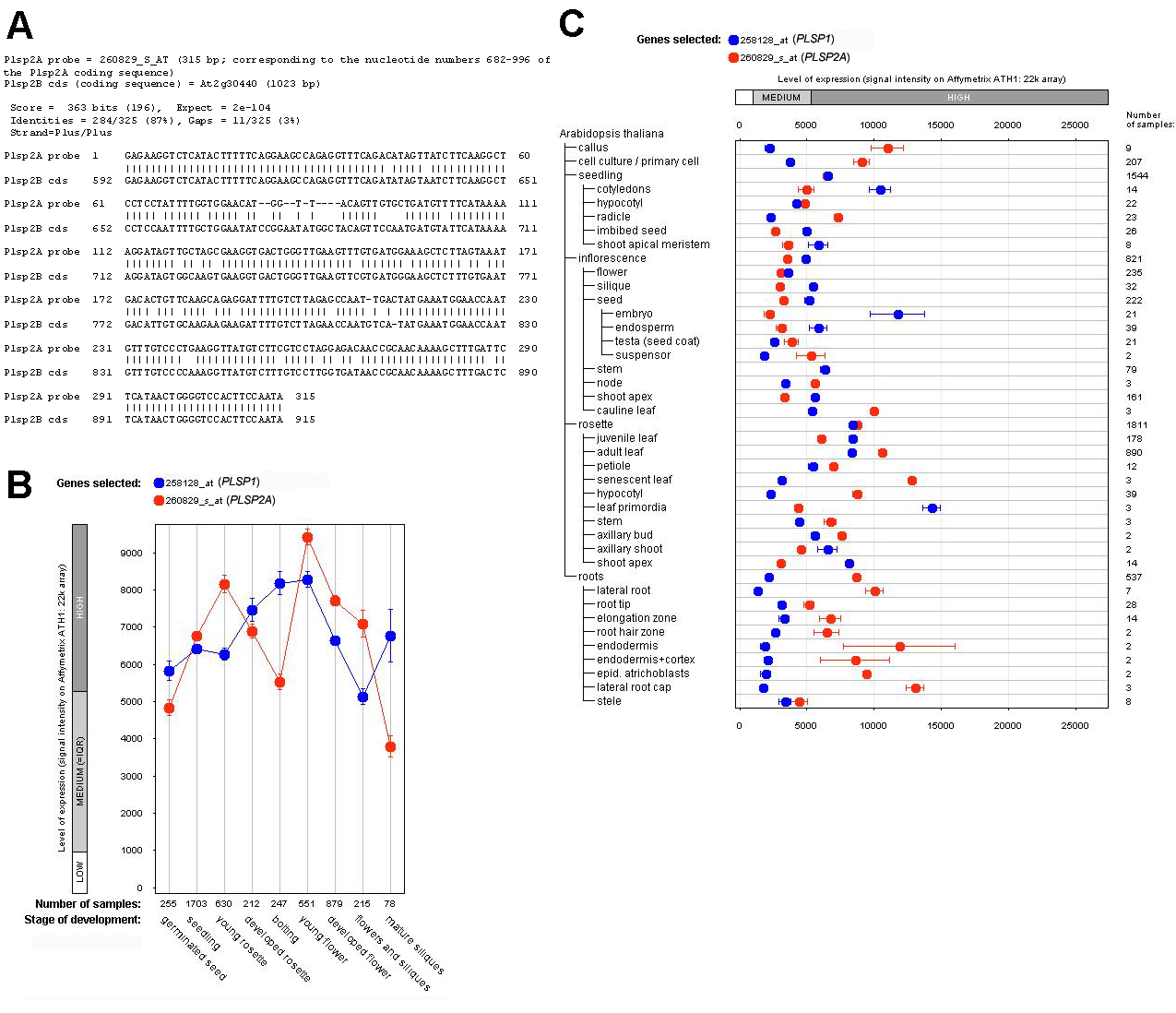

Supplement: Figure S4 — A) Alignment of the PLSP2A probe and the PLSP2B sequences. B) In silico data for the expression of TPP genes in A. thaliana according to the development stages of the plants. C) In silico data for the expression of TPP genes in A. thaliana according to different tissue types. (TIF) [file pone.0027258.s004.tif]

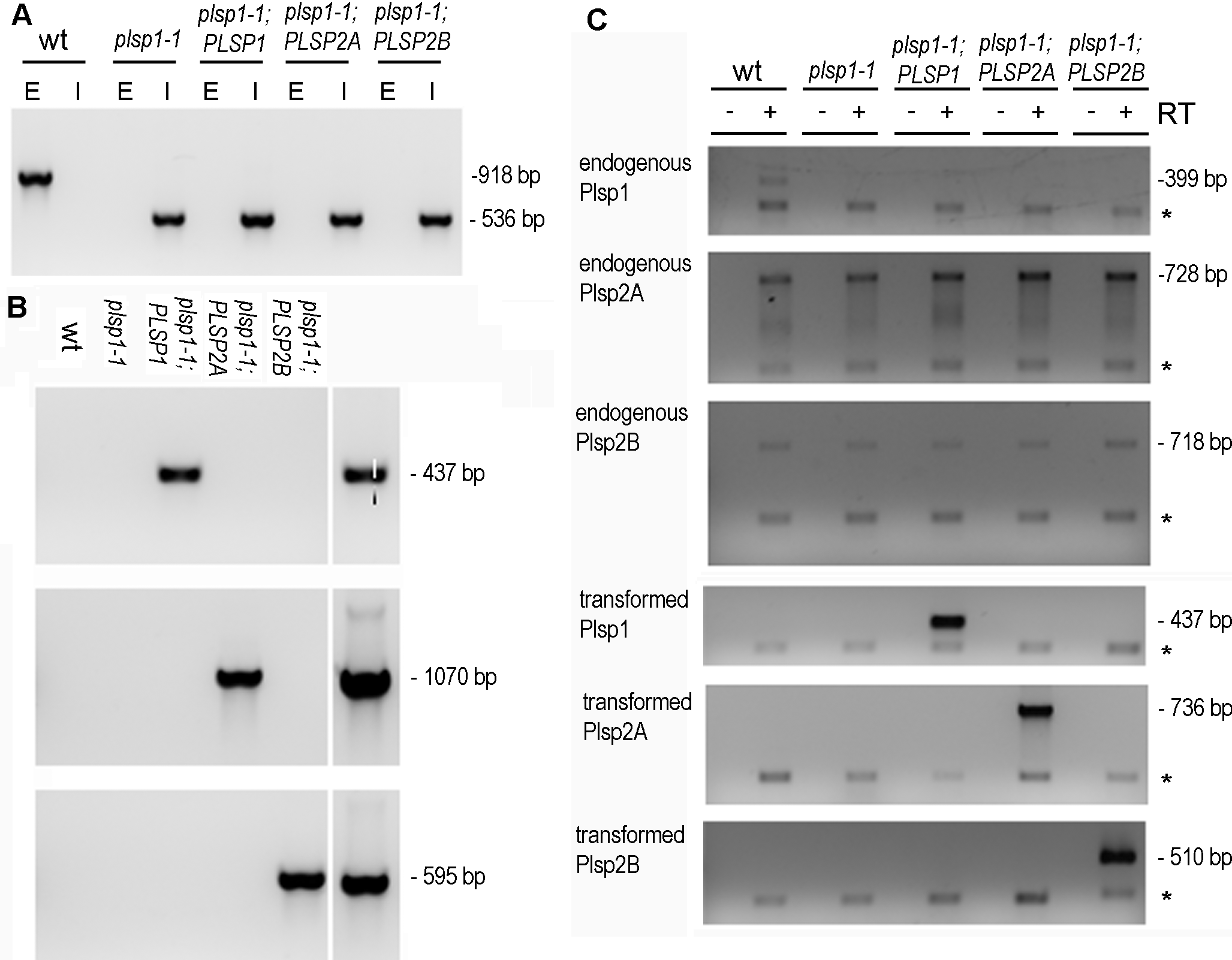

Supplement: Figure S5 — A) Genomic PCR of wild-type (wt) and mutant A. thaliana seedlings. E and I indicate reactions specific to amplify the inserted T-DNA into PLSP1 (918 bp) and part of the endogenous PLSP1 (536 bp). B) Genomic PCR of wild-type and mutant A. thaliana seedlings. Presented are reactions specific to amplify the transgene introduced into the plsp1-null mutant encoding Plsp1 (437 bp), Plsp2A (1070 bp), and Plsp2B (595 bp). Far right lanes show the reactions using the plasmid used for transformation. C) RT-PCR profiles of wild-type and mutant A. thaliana seedlings for genes indicated at left. Each reaction contained two sets of primers: one for each cDNA whose size is indicated at right, and another for cDNA derived from 18S RNA indicated with an asterisk. The template used was either total RNA without (−) or with reverse transcription (+, RT). (TIF) [file pone.0027258.s005.tif]
