# Supplementary material for: Functional Diversification of Thylakoidal Processing Peptidases in Arabidopsis thaliana
Source: PLoS One. 2011 Nov 7;6(11):e27258. doi: 10.1371/journal.pone.0027258 (PMC3210150; doi:10.1371/journal.pone.0027258)
Supplement: Table S1 — Sequences of oligonucleotide primers used in this study. All sequences are depicted from 5′ to 3′. F and R indicate forward and reverse primers, respectively. (DOC) [file pone.0027258.s006.doc]

**Table S1.** Sequences of oligonucleotide primers used in this study

All sequences are depicted from 5’ to 3’. F and R indicate forward and reverse primers, respectively.

| **cDNA Cloning into pGEM®-T Easy vector** | |
| --- | --- |
| Plsp2A | F: CATTTATGGCGATTAGGGTTACCT  R: ATACCATCTGCGTTGGTCAAGATA |
| **cDNA Cloning into the pET16b-Kan vector** (introduced *Bam*HI sites are indicated with underlines) | |
| Plsp1 | F: C**GGATCC**AAGATATATTCCTTCT  R: G**GGATCC**TATTGCTTATCCACAGCA |
| Plsp2A | F: C**GGATCC**AAAGTCTATACCTTCA  R: G**GGATCC**TCAAGATACATCAACAGCTCT |
| **qRT-PCR** |  |
| *PLSP1* | F: ATTCGCACGTATGGGTCCTC  R: TGTCCCGCTTACTCTGTTTGGT |
| *PLSP2A* | F: GTTGAATATCTGCTCGGAGGA  R: TCACAAACTTCAACCCAGTCAC |
| *PLSP2B* | F: GCTCCTCCAATTTTGCTGGAA  R: AAACTGCAACAGGTCCCCTTG |
| *PP2A1* | F: CAAGAGGTTCCACACGAAGGA  R: TGTAACCAGCACCACGAGGA |
| **Genotyping of the *plsp1-1* mutant** | |
| *PLSP1*^a^ | F: AACGGATTGTTGCCAAAGAAGG  R: GCAGCTTCCGACAAGAAGGGT |
| Salk LBa_1 | GCGTGGACCGCTTGCTGCAACTC |
| **cDNA Cloning into the pMDC32 vector** | |
| *attb1* adapters | F: GGGGACAAGTTTGTACAAAAAAGCAGGCTCGCCCATG  R: GGGGACCACTTTGTACAAGAAAGCTGGGTTTA |
| *PLSP1* | F: CAAAAAAAGCAGGCTCGCCCATGATGGTGATGATATCTC  R: GTACAAGAAAGCTGGGTCCTATTGCTTATCCACAGCAC |
| *PLSP2A* | F: CAAAAAAAGCAGGCTCGCCCATGGCGATTAGGGTTACC  R: GTACAAGAAAGCTGGGTCTCAAGATACATCAACAGC |
| *PLSP2B* | F: CAAAAAAAGCAGGCTCGCCAATGGCGATAAGAATC  R: GTACAAGAAAGCTGGGTCTCATGAAACTGCAACAGG |
| **RT-PCR** |  |
| Endogenous *PLSP1* | F^b^: AACGGATTGTTGCCAAAGAAGG  R: CGGATCCCATTAATCTGCAATCTAATTC |
| Endogenous *PLSP2A* | F: CCTCGAGACAACAAGAAGAAGAAGAC  R: CATCGATCAACTCCAACTTCACTTTAGG |
| Endogenous *PLSP2B* | F: CTTGTTCAAGTTCTTCATC  R: GTAGCCATATTCCGGATA |
| Transgene for Plsp1^b^ | F^b^: AACGGATTGTTGCCAAAGAAGG |
| Transgene for Plsp2A | F: GTTGAATATCTGCTCGGAGGA |
| Transgene for Plsp2B | F: GCTCCTCCAATTTTGCTGGAA |
| *nos* terminator primer | R: AGACCGGCAACAGGATTCAATC |

^a^Also used in Inoue et al. 2005.

^b^Also used for *PLSP1* genotyping.
